# 

**Published:** 1924-11

**Authors:** 


					New Series. Vol. III. Wo. 38 (V^.L?,11). November, 1924. Monthly, Price 6d. (pos?E

				

